# Validation of a Simple and Reliable Method for the Determination of Aflatoxins in Soil and Food Matrices

**DOI:** 10.1021/acsomega.1c01451

**Published:** 2021-07-16

**Authors:** Julius Albert, Camilla A. More, Niklaus R. P. Dahlke, Zacharias Steinmetz, Gabriele E. Schaumann, Katherine Muñoz

**Affiliations:** † iES Landau, Institute for Environmental Sciences, Group of Organic and Ecological Chemistry, University of Koblenz-Landau, Fortstraße 7, 76829 Landau, Germany; ‡ iES Landau, Institute for Environmental Sciences, Group of Environmental and Soil Chemistry, University of Koblenz-Landau, Fortstraße 7, 76829 Landau, Germany

## Abstract

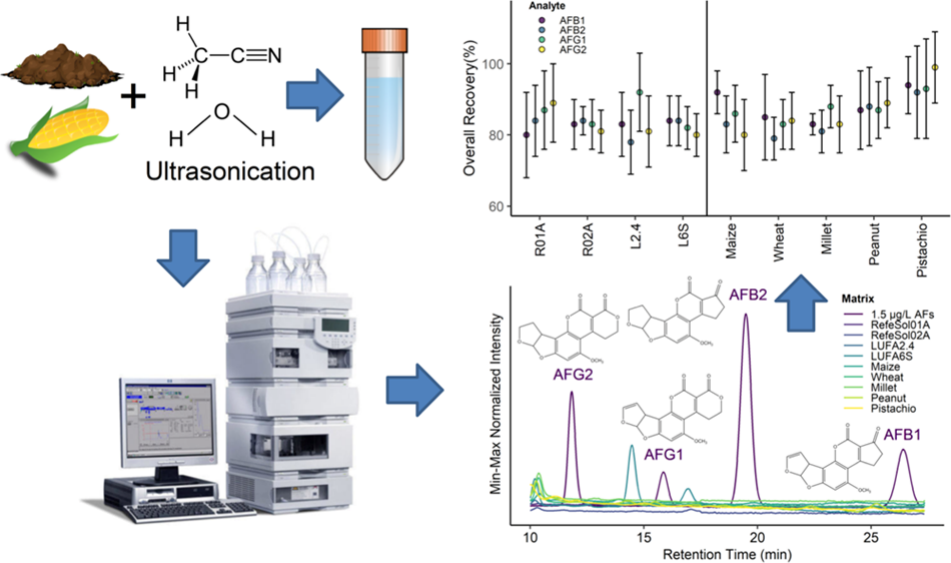

Aflatoxins (AFs) are toxic fungal secondary metabolites that are commonly detected in food commodities. Currently, there is a lack of generic methods capable of determining AFs both at postharvest stages in agricultural products and preharvest stages, namely, the agricultural soil. Here, we present a simple and reliable method for quantitative analysis of AFs in soil and food matrices at environmentally relevant concentrations for the first time, using the same extraction procedure and chromatography, either by HPLC-FLD or LC–MS. AFs were extracted from matrices by ultrasonication using an acetonitrile/water mixture (84:16, v + v) without extensive and time-consuming cleanup procedures. Food extracts were defatted with *n*-hexane. Matrix effects in terms of signal suppression/enhancement (SSE) for HPLC-FLD were within ±20% for all matrices tested. For LC–MS, the SSE values were mostly within ±20% for soil matrices but outside ±20% for all food matrices. The sensitivity of the method allowed quantitative analysis even at trace levels with quantification limits (LOQs) between 0.04 and 0.23 μg kg^–1^ for HPLC-FLD and 0.06–0.23 μg kg^–1^ for LC–MS. The recoveries ranged from 64 to 92, 74 to 101, and 78 to 103% for fortification levels of 0.5, 5, and 20 μg kg^–1^, respectively, with repeatability values of 2–18%. The validation results are in accordance with the quality criteria and limits for mycotoxins set by the European Commission, thus confirming a satisfactory performance of the analytical method. Although reliable analysis is possible with both instruments, the HPLC-FLD method may be more suitable for routine analysis because it does not require consideration of the matrix.

## 1 Introduction

Aflatoxins (AFs) are secondary metabolites produced by certain molds of the genus *Aspergillus* that are widespread in crops and food commodities. AFs are toxic and carcinogenic to humans and therefore, their occurrence is associated with serious health concerns. As a consequence, maximum limits have been set in foods for the main AFs B1 (AFB1), B2 (AFB2), G1 (AFG1), and G2 (AFG2) in order to protect consumers against dietary exposure. Commodities exceeding the maximum levels cannot be further commercialized, leading to substantial economic losses for agriculture and livestock farmers. At present, AFs are almost exclusively studied for their potential to contaminate food and feed, which is reflected in the overwhelming research on AFs and aflatoxigenic fungi with regard to their chemistry, and the causes of their occurrence in feed and food and to the toxic effects that they may exert on humans and animals.^1^ In order to understand the environmental occurrence and fate of AFs, suitable and reliable analytical methods are required. These methods should be accessible not only at the level of the agricultural product but also considering previous steps in the production of commodities, namely, the plant–soil ecosystem. Soil is considered the natural habitat for aflatoxigenic fungi and serves as a reservoir for primary inoculum in plant infestation.^2^ For this reason, the development and validation of analytical tools which investigate the potential of soil as a mycotoxin source are imperative.

The contamination levels of AFs reported in agricultural soils ranged from 10^–2^ to 10^1^ μg kg^–1^.^4^ These levels however may not represent environmental concentrations since the described analytical method has not been subjected to a systematic validation in terms of sensitivity, accuracy, and matrix effects. Other presented recovery rates for soil matrices were either not suitable^4,5^ or the procedures were not systematically validated^3,6,7^ (Table 1). In addition, AF fortification levels were in the range of 10–10^5^ μg kg^−1^,^4,5,8,9^ which may be far above the environmentally relevant levels (Table 1). A probable reason for the lack of validation proceeding may be attributed to the complexity of soil as the environmental matrix and to the interaction of AFs with soil fractions.^1^ In this context, the soil organic matter and soil texture are of particular importance since AFs strongly sorb to soil organic carbon^10,11^ and clay minerals.^4,5,12,13^ This methodological challenge may be overcome by weakening the chemical interactions between the matrix and AFs via introduction of additional extraction procedures to further facilitate the transition of the analytes into the liquid phase. The introduction of an additional ultrasonication step during solvent extraction (USE) is reported to minimize solvent consumption while improving the extraction efficiency for many substances.^14^ As far as we know, a USE method for the extraction of AFs from soil matrices has not yet been reported. However, due to the limited selectivity of USE, a high load of matrix components is simultaneously extracted with the analytes. Such coextracted matrix components can heavily affect the analytical performance of the detection method, which is why USE methods are often used in combination with further cleanup steps.^14^ For analysis of AFs, liquid chromatography with mass spectrometry (LC–MS) and fluorescence detection (HPLC-FLD) are the methods of choice.^15,16^ Both methods are however prone to interferences with coeluting matrix components, affecting both the separation step and the intensity of the detection response. In case of LC–MS, such coeluting matrix components strongly affect the ionization efficiency of the target analytes, resulting in either a loss or an increase in response. This matrix effect must be evaluated when validating a method to avoid over- or underestimation of the concentration.^17^ If such matrix components also emit fluorescence at the wavelengths of the target analytes and are not sufficiently separated from the target peaks, such coeluting matrix components may cause a false-positive result. Current methods used in AF analytics to overcome matrix interactions and effects are solid-phase extraction with silica gels, imprinted polymers, or immunoaffinity columns.^18^ However, these methods are associated with a comparatively high cost and workload, particularly in routine analysis of environmental samples. The current strong dependence of AF analysis on extensive and expensive sample purification techniques or on analytical tools such as LC–MS is a problem, particularly in countries affected by AF outbreaks.^16^ Hence, there is a need for cost-efficient and simple alternative approaches.

**Table 1:** Previously Described Methods for the Extraction of AFs from Soil Samples^a^

extraction technique	solvents	extraction procedure	soil type	clay (%)	*C*_org_ (%)	fortification level (μg kg^–1^)	recovery (%)	references
solvent extraction	acetone	30 min shaking	silt loam	22.2	2.4	1 × 10^4^	18	Angle & Wagner^5^
solvent extraction	chloroform, MeOH, chloroform/MeOH (80:20)	NA	loam soil	28.1	NA	3.3–26.7 × 10^4^	<1	Mertz et al.^4^
solvent extraction	acetone	5 min blending	silt loam	33.6	2.9	5.7 × 10^3^	70	Goldberg & Angle^9^
			sandy loam	12.1	1.5			
			clay loam	27.5	1.8			
			silty clay loam	37.8	0.6			
supercritical fluid extraction	acetonitile +2% acetic acid	15 min static time	silt loam	58.5	1.87	1.7 × 10^3^	72	Starr & Selim^8^
solvent extraction	water/ethyl acetate (1:3)	overnight shaking	silt loam	8.1–8.3	0.47–0.55	10	NA	Accinelli et al.^7^

a NA = not available, *C*_org_ = soil organic carbon content.

In the present work, the suitability of a generic and proven solvent composition (acetonitrile/water, 84/16, v + v)^19−22^ in combination with ultrasonication for the extraction of AFs from soils and plant-based foods is tested and validated according to the requirements of the Eurachem Guide^23^ and European Commission (EC) Regulation no. 401/2006.^24^ We aimed to prevent the coelution of interfering peaks by developing a suitable chromatographic method to enable analysis without extensive and time-consuming cleanup steps. To evaluate the effect of matrix composition, four different agricultural soils and five agricultural products were evaluated using USE, followed by LC–MS analysis. The optimized procedure was evaluated in terms of recovery, linearity, selectivity, precision, detection and quantification limits (LOD and LOQ, respectively), and matrix effects.

## 2 Experimental Section

### 2.1 Chemicals and Reagents

Methanol (MeOH) and acetonitrile (MeCN) used for extraction, HPLC-FLD chromatography and preparation of standards were of the HPLC grade (Carl Roth, Karlsruhe, Germany). MeOH for LC–MS chromatography was of the LC–MS grade (Fisher Scientific, Schwerte, Germany). Ultrapure water (H_2_O) was used throughout all work and was produced by a Milli-Q-water purification system (18.2 MΩ cm^–1^, EASYpure II, Millipore Bedford, MA). A standard mix solution with certified concentrations of 20 μg mL^–1^ each for AFB1, AFB2, AFG1, and AFG2 dissolved in MeCN was purchased from Sigma-Aldrich (Sigma-Aldrich, St. Louis, USA). From this standard solution, a working standard solution of an AF mixture containing 1000 μg L^–1^ of each AF was prepared in MeOH. This working standard was used for fortification of samples and preparation of calibration standards (solvent and matrix matched) in MeOH/H_2_O (20:80, v + v). All solutions were stored at −20 °C in the dark until analysis.

### 2.2 Soil and Food Samples

Experiments were carried out using four soil types and five different food commodities to compensate for differences in matrices. RefeSol 01-A and RefeSol 02-A (Fraunhofer IME, Schmallenberg, Germany) and LUFA 2.4 and LUFA 6S (LUFA, Speyer, Germany) served as reference soils from organically managed arable areas. These soils were selected to cover a wide range of physicochemical properties, which are expected to have an influence on extraction efficiency (Table 2). The soil organic carbon and clay mineral contents, as reflected in soil texture (clay content), are of particular interest as these soil fractions represent sorption sites for organic molecules and can thus impede successful extraction.^4,5,10−13^ Soils were homogenized, air-dried, and 2 mm-sieved. The five selected food matrices included maize, wheat, millet, peanut, and pistachio. These foods were selected because of their relevance as commodities frequently contaminated by AFs. Matrices were obtained as powders at a local retail market except for pistachios and peanuts, which were mechanically ground to obtain a fine and homogenized powder prior to the extraction. All food samples were air-dried prior to extraction.

**Table 2:** Physicochemical Properties of the Tested Reference Soils^a^

soil	sand (%)	silt (%)	clay (%)	*C*_org_ (%)	pH	CEC (mequiv/100 g)
RefeSol 01-A	74	19.8	6.2	0.89	5.3	1.16
RefeSol 02-A	5.7	78.3	16.0	1.04	6.6	12.5
LUFA 2.4	32.1	41.6	26.3	1.78	7.4	24.2
LUFA 6S	23.8	35.3	40.9	1.99	7.2	23

a *C*_org_ = soil organic carbon content, CEC = cation exchange capacity.

### 2.3 Sample Fortification and Extraction

Fractions of 5 g (dry weight, dw) of air-dried samples were placed in 50 mL centrifuge tubes and fortified with AFB1, AFB2, AFG1, and AFG2. Three contamination levels (0.5, 5, and 20 μg kg^–1^) were achieved via fortification with 100 μL of 0, 25, 250, and 1000 μg L^–1^ in methanolic solutions. Fortification levels were chosen to fit with the concentration levels set by Commission Regulation (EC) no. 401/2006^24^ (i.e., ≤1 μg kg^–1^, 1–10 μg kg^–1^, and >10 μg kg^–1^), which are used to evaluate the suitability of an analytical method in terms of recovery rates. The three fortification levels were compared with extraction without fortification. Fortified samples were vortexed for 10 s to obtain a homogeneous sample and left under the fume hood for 30 min to allow the solvent to evaporate. AFs were extracted from the samples using 15 mL of a MeCN/H_2_O (84:16, v + v) mixture using an orbital shaker at 180 rpm for 30 min. The extraction was followed by 15 min ultrasonication followed by centrifugation at 2190*g* for 5 min. A 1 mL aliquot was transferred to centrifuge tubes and evaporated until dryness under a gently stream of nitrogen at 40 °C. The dried extracts were reconstituted with 200 μL of MeOH and vortexed for 10 s. The reconstituted samples were then conditioned with 800 μL water and vortex-mixed for 10 s. Aliquots of 400 μL of *n*-hexane was added to the extracts obtained from food matrices and vortexed for 10 s to remove the coextracted fat,^25,26^ which may otherwise negatively affect the analytical performance.^27−29^ The *n*-hexane layer was discarded. To remove undissolved particles, the conditioned samples were centrifuged at 13,000*g* for 1 min and the supernatant was transferred to HPLC amber glass vials. The filtered extracts were stored at −20 °C in dark until measurements.

### 2.4 LC–MS Analysis

LC–MS analyses were performed on an Exactive Orbitrap system (ThermoFisher Scientific Inc., Waltham, USA) operating in positive mode in the range of 200–500 *m*/*z*. The scan was performed in high-resolution mode corresponding to a value of 50,000 at *m*/*z* 200 at a scan rate of 2 Hz. The automatic gain control (AGC) target value was set to 1 × 10^6^ (balanced). By foregoing exhaustive extract purification for matrix removal, an Orbitrap system allows for higher-resolution detection of *m*/*z* ratios. AFs were separated on a Hypersil GOLD C18 1.9 μm 1.0 × 100 mm column (ThermoFisher Scientific Inc., Waltham, USA) by gradient elution using MeOH (eluent A) and ultrapure water (eluent B), both conditioned with 0.1% formic acid and 4 mM ammonium formate at a flow rate of 0.2 mL min^–1^. The program (i) began with an isocratic phase of 2 min at 10% eluent A, (ii) followed by a linear increase to 95% over 8 min, (iii) an isocratic phase of 3 min, (iv) a linear decrease to 10%, and (v) a reconditioning phase of 2 min. The injection volume was set at 10 μL for both sample extracts and calibration standards. AFs were measured in positive electrospray ionization mode with [M + H]^+^ adducts. Target analysis was performed with ionic masses at 313.0715, 315.0860, 329.0650, and 331.0800 *m*/*z* for AFB1, AFB2, AFG1, and AFG2 respectively. Furthermore, the [M + NH_4_]^+^ adduct was continuously monitored alongside the [M + H]^+^ adduct for confirmation purposes, with *m*/*z* of 330.0962, 332.1132, 351.0467, and 353.0631 for AFB1, AFB2, AFG1, and AFG2, respectively. A concentration-to-signal relationship for the [M + NH_4_]^+^ adduct, as well as the absence of a signal in the matrix blank was confirmed (Figure S2). The electronic setting was defined as follows: capillary voltage, 25 V; spray voltage, 4 kV; tube lens voltage, 75 V; skimmer voltage, 14 V; capillary temperature, 275 °C.

### 2.5 HPLC-FLD Analysis

The AF analyses were performed on an Agilent 1200 series (Agilent, Santa Clara, USA) system (G1311A Quaternary pump, G1322A degasser, G1329A autosampler) equipped with a column oven (Jetstream 2 column thermostat, KNAUER, Berlin, Germany), postcolumn UV-derivatization module (UVE, KNAUER, Berlin, Germany), and fluorescence detector (G1321A, Agilent, Santa Clara, USA). Chromatographic separation of AFs was achieved on a Zorbax Eclipse XDB-C18 reversed-phase 5 μm 4.6 × 150 mm column (CS Chromatographie-Service, Langerwehe, Germany) using an isocratic elution mode consisting of a mixture of H_2_O/MeOH/MeCN (72:20:8, v + v + v) at a flow rate of 1.7 mL min^–1^. The injection volume was set at 100 μL for both sample extracts and calibration standards. The fluorescence detector was set to an excitation wavelength of 365 nm and emission wavelengths of 455 nm for AFG1 and AFG2 and 435 nm for AFB1 and AFB2. The selection criteria for AFs in samples were the retention time and peak shape of the analytes observed in matrix-matched calibration solutions.

### 2.6 Quality Criteria

The method was tested in terms of the selectivity, linear working range, matrix effects, accuracy (trueness and precision), LOD, and LOQ in accordance with the EuraChem guide^23^ to fulfill the requirements of Commission Regulation (EC) no. 401/2006.^24^

The selectivity was tested through (i) the analysis of nonfortified and fortified samples at four levels (no spike, 0.5, 5, and 20 μg kg^–1^) via LC–MS and HPLC-FLD, (ii) matrix-matched calibration standards via LC–MS and HPLC-FLD, and (iii) identification of alleged AF peaks via *m*/*z*-ratios of adduct with the highest ([M + H]^+^) intensity as the quantifier and the second highest intensity ([M + NH_4_]^+^) as the qualifier using high-resolution MS detection.

The linear working range was assessed through measurements of 10 calibration levels in the range of 0.01–50 μg L^–1^. Nominal concentrations were plotted against the integrated area. A linear range between 0.05 and 10 μg L^–1^, equivalent to 0.15–30 μg kg^–1^ dry solid matrix, was approximated by visual inspection of the scatter plot.^30^ Linearity of the approximated working range was estimated by duplicate measurements of six calibration standards in the range between 0.05 and 10 μg L^–1^ by the use of residual plots (residuals vs predicted values) and calculation of the adjusted coefficient of determination (*R*^2^_adj_).^30^ The assumption of normality was assessed via QQ-plots (standardized residuals vs theoretical quantiles).^30^ The homoscedasticity criterion was checked via scale-location plots (square root of standardized residuals vs predicted values).^30^ In case calibration data did not meet the assumption of homoscedasticity, the weighted least-squares (WLSs) linear regression model was applied as a simple and effective way to counteract the greater influence of higher concentrations on the regression model, improving the accuracy at the lower end of the calibration curve.^31^ The optimal weighting factor *w*_*i*_ was chosen according to the procedure described by Almeida.^31^ The following *w*_*i*_ were tested: 1/*x*^0.5^, 1/*x*, 1/*x*^2^, 1/*y*^0.5^, 1/*y*, and 1/*y*^2^, where *x* is the nominal concentration and y is the signal (i.e. peak area). In brief, the best weighting factor was chosen according to the percentage relative error (% RE), which compares the calculated concentrations *x*_calc_ with the nominal concentrations *x* for all tested weighted models
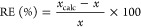


The best *w*_*i*_ was that which presents the least RE_sum_ (%)
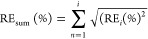


The magnitude of matrix effects was estimated by comparing the slopes of solvent (*b*_sol_) and matrix-matched calibrations (*b*_mm_) and quantitatively expressed as the signal suppression/enhancement (SSE) ratio using the following equation^32^
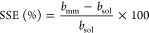


All AF concentrations were calculated using weighted matrix-matched calibration.

Trueness in terms of bias was calculated as relative spike recovery *R* (%) using data from spiking experiments with the following equation
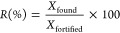
where *X*_found_ is the concentration calculated using the weighted matrix-matched calibration curve and *X*_fortified_ is the nominal added concentration. According to Commission Regulation (EC) no. 401/2006,^24^ recovery rates for AFs should be in the range of 50–120, 70–110, and 80–110% for concentrations ≤1, 1–10, and >10 μg kg^–1^, respectively.

Precision in terms of repeatability was estimated as the relative standard deviation (RSD_r_ (%)) of replicate measurements at each fortification level (*n* = 10)
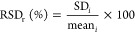
where SD_*i*_ is the standard deviation and mean_*i*_ is the arithmetic mean of respective recovery rates. According to Commission Regulation (EC) no. 401/2006,^24^ the recommended maximum relative standard deviation under repeatable conditions RSD_r,rec_ can be calculated using following equation
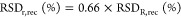
where RSD_R,rec_ is the recommended maximum relative standard deviation under reproducible conditions, which can be derived from the modified Horwitz equation^33^ for concentration ratios <1.2 × 10^–7^ (i.e., 1 = 100 g/100 g, 0.001 = 1000 mg/kg)
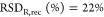


This results in RSD_r,rec_ of 14.52% for the concentrations studied. However, according to Commission Regulation (EC) no. 401/2006,^24^ the maximum permitted relative standard deviation may be double the recommended value, that is, 29.04%.

The LOD and LOQ were estimated based on data of the recovery experiment. Samples fortified with 0.5 μg kg^–1^ AFs (*N* = 10) were used for determination of the LOD and LOQ. The absence of AFs was previously confirmed for the investigated soil and food samples. The LOD and LOQ were calculated using the following equations
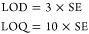
where SE is the sample standard error derived from replicate observations. The target quantification level is set to the maximum levels for certain contaminants in foodstuff intended for direct human consumption listed in Commission Regulation (EC) no. 1881/2006.^24^ In brief, these limits are as follows: 2 (AFB1) and 4 μg kg^–1^ (sum AFB1, AFB2, AFG1, AFG2) for peanut, maize, wheat, millet; 8 (AFB1) and 10 μg kg^–1^ (sum AFB1, AFB2, AFG1, AFG2) for pistachio; 0.1 μg kg^–1^ (AFB1) for baby, infant, young children, and medical use. However, for soil matrices, no limits are defined yet by the EC.

### 2.7 Data Analysis

Data processing and statistical analyses were performed using R (version 4.0.3, R Core Team). The weighting factors for weighted calibration were selected based on the minimum RE_sum_ using the command “weight_select” (package “envalysis”, available from https://doi.org/ft9p). Matrix effects in terms of SSE were calculated using the command “matrix_effect” (package “envalysis”, available from https://doi.org/ft9p). Effects of the matrix type (“Matrix type”; factor with the two levels “food” and “soil”) and instrumentation (“Method”; factor with two levels “LC–MS” and “HPLC-FLD”) and their interaction on the absolute value of the matrix effect (SSE), LOD, and LOQ were tested using two-way ANOVA models. Effects of the matrix type (“Matrix type”; factor with the two levels “food” and “soil”) and fortification level (“Fortification level”; factor with three levels “low”, “medium” and “high”) and their interaction on recovery (Recovery) and relative standard deviation (RSD_r_) were tested using two-way ANOVA models. The significance of predictor variables was tested with an F-test. The effect of clay content (“clay”) and soil organic carbon content (*C*_org_) on recovery (“Recovery) was tested via linear mixed effect models with the command “lmer” (package “lmerTest”, available from https://doi.org/dg3k). Because of the nested design, where recovery rates are obtained at different fortification levels, the variable “fortification level” (factor with the three levels “low”, “medium” and “high”) was included as a random effect. Kenward–Roger approximation was used for computing the degrees of freedom and *t*-statistics of the predictors of the mixed effect models (package “lmerTest”, available from https://doi.org/dg3k).^34^ Significant results (*P* < 0.05) are shown in bold. Model assumptions were verified using diagnostic plots, that is, normality of residuals was checked via QQ-plots and homoscedasticity of residuals was checked via scale-location plots (square root of standardized residuals vs predicted values).^30^

## 3 Results and Discussion

### 3.1 HPLC-FLD Method Development

**Figure 1:**
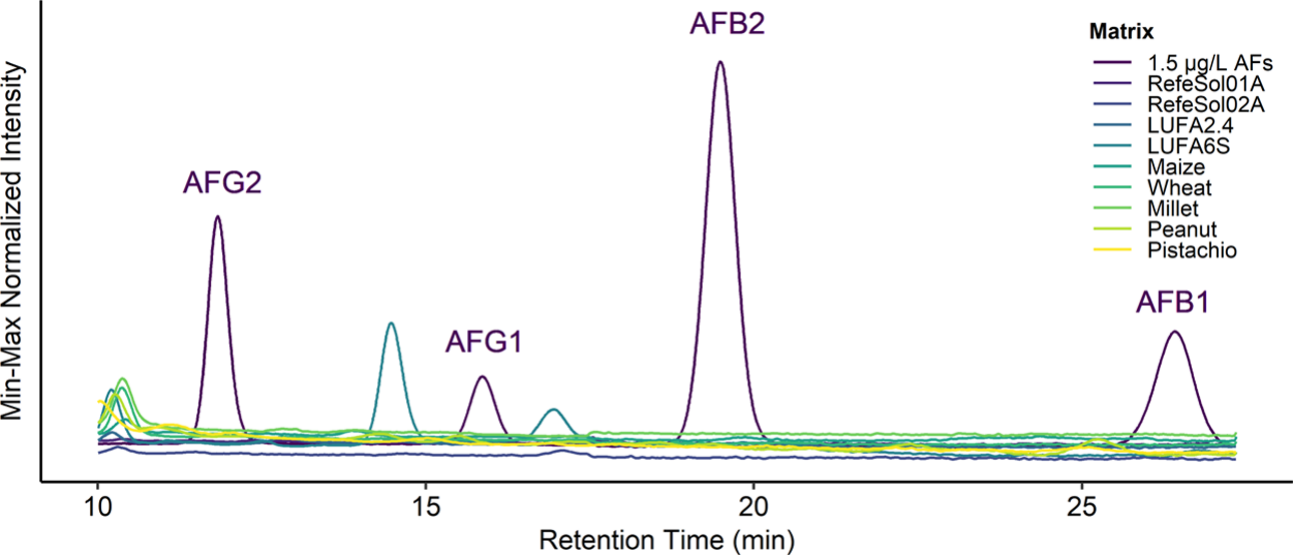
Extracted HPLC-FLD chromatograms obtained from injection of the solvent calibration standard at 1.5 μg L^–1^ and blanks of respective matrices (highlighted by different colors). Additional chromatograms showing sample blanks, solvent calibration standard (1.5 μg L^–1^), matrix-matched calibration standard (1.5 μg L^–1^), and fortified sample extracts (20 ng g^–1^) for two food matrices (maize and wheat) and two soil matrices (RefeSol 01-A, LUFA 6S) are presented in the Supporting Information (Figure S1).

During development of the HPLC-FLD method, several chromatographic conditions were tested such as different combinations of H_2_O/MeOH/MeCN at different column temperatures. Frequently used eluent mixtures at 55–65% H_2_O and variable amounts of MeOH and MeCN^35−39^ achieved baseline separation of the four investigated AFs. However, none of the tested conditions were able to baseline-separate interference peaks from the analyte peaks. To overcome the coelution problem, weaker mobile-phase compositions in terms of elution power were tested at different temperatures and flow rates. An increase in the H_2_O content prolonged the run time but resulted in better separation. An increase in temperature and flow rate lowered the run time but lead to insufficient separation and a decrease in the sensitivity. Furthermore, the ratio between MeOH and MeCN considerably hampered the separation of AFG1 and AFB2. While an increase in MeCN generally resulted in faster run time, it led to poor baseline separation of the AFG1 and AFB2 peaks. Finally, a separation at 35 °C and a mobile-phase composition of 72:20:8 (v + v + v) H_2_O/MeOH/MeCN at a flow rate of 1.7 mL min^–1^ proved to be a good compromise between separation performance, speed, and sensitivity. Chromatograms of all tested matrices for the optimized method are presented in Figure 1. Additional chromatograms for sample blanks, solvent, and matrix-matched calibration standard (1.5 μg L^–1^) and fortified sample extracts (20 ng g^–1^) for two food matrices (maize and wheat) and two soil matrices (RefeSol 01-A and LUFA 6S) are available in the Supporting Information (Figure S1).

### 3.2 Matrix Effects, Interferences, and Linear Working Range

**Figure 2:**
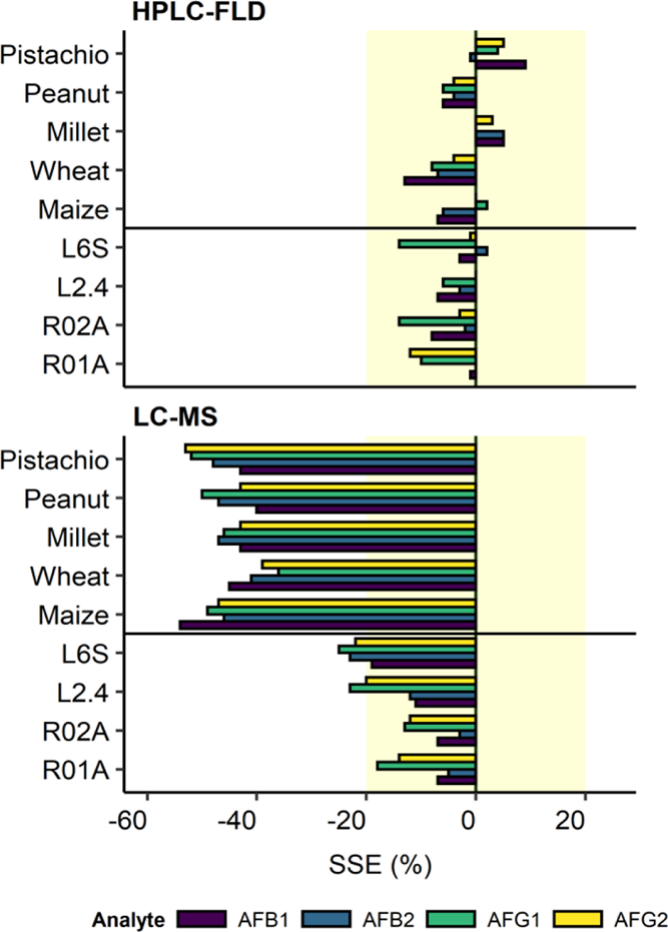
Matrix effects in terms of SSE for HPLC-FLD (left) and LC–MS (right). The colored band marks the threshold of ±20% to justify using solvent calibration, as opposed to the matrix-matched standard.

Prior to the analysis of fortified samples, linear working range, matrix effects, and necessity of weighting were evaluated for all analytes in all matrices in order to determine the quantification strategy. Suitable *R*_adj_^2^ ≥ 0.991 were achieved for all calibration curves in all matrices and for all analytes using the nonweighted calibration (Table S1). However, since all calibration models did not meet the assumption of homoscedasticity, WLS models were applied to improve the precision at the lower end of the calibration. Although the *R*_adj_^2^ values had significantly decreased by an average of −0.0072 (*p* < 0.001, paired *t*-test, d*f* = 79, Tables S1 and S3), the application of w_i_ significantly reduced the RE_sum_ (%) by an average of −284% (*p* < 0.001, paired *t*-test, d*f* = 79, Tables S1 and S3) and hence improved the precision at the lower end of the calibrations. The slope ratio of the weighted matrix matched and solvent calibration was then used to evaluate matrix effects. Matrix effects in terms of the SSE were significantly (*p* < 0.001, F-ANOVA, d*f* = 1, Tables S1 and S3) lower for the HPLC-FLD (average 5 ± 4%) than for the LC–MS (average 31 ± 8%) method (Figure 2). Moreover, the interaction between instrument and matrix type was significant (*p* < 0.001, F-ANOVA, d*f* = 1, Tables S1 and S3), indicating stronger matrix effects in food matrices than in soil matrices for LC–MS, while the opposite pattern was observed for HPLC-FLD (Figure 2).

SSE values ±20% are generally considered suitable values, indicating minor matrix effects and may function as a threshold to justify using solvent calibration as opposed to matrix-matched calibration as this variation would be close to repeatability values.^40,41^ Overall, the matrix effects were within the ±20% range for all matrices tested via HPLC-FLD. Furthermore, matrix effects were mostly within the 20% threshold for the soil matrices tested via LC–MS. Thus, according to the suggested threshold of ±20%, it would be sufficient to use solvent calibration instead of matrix-matched calibration for concentration calculation. Since the use of a matrix-matched calibration would require an analyte-free matrix blank, the possibility of using a solvent calibration instead of a matrix-matched calibration makes the proposed method applicable to cases where no sample blank is available. All food matrices tested via LC–MS were far below the threshold of ±20%, and hence sample purification (i.e., immunoaffinity chromatography (IAC) or solid-phase extraction (SPE) procedures) or matrix-effect compensation strategies such as matrix-matched calibration and stable isotope dilution assays would be necessary.^42^ In contrast, since no coeluting interferences occurred and matrix effects were almost negligible, HPLC-FLD may be more suitable for routine analyses.

### 3.3 Limits of Detection and Quantification

**Figure 3:**
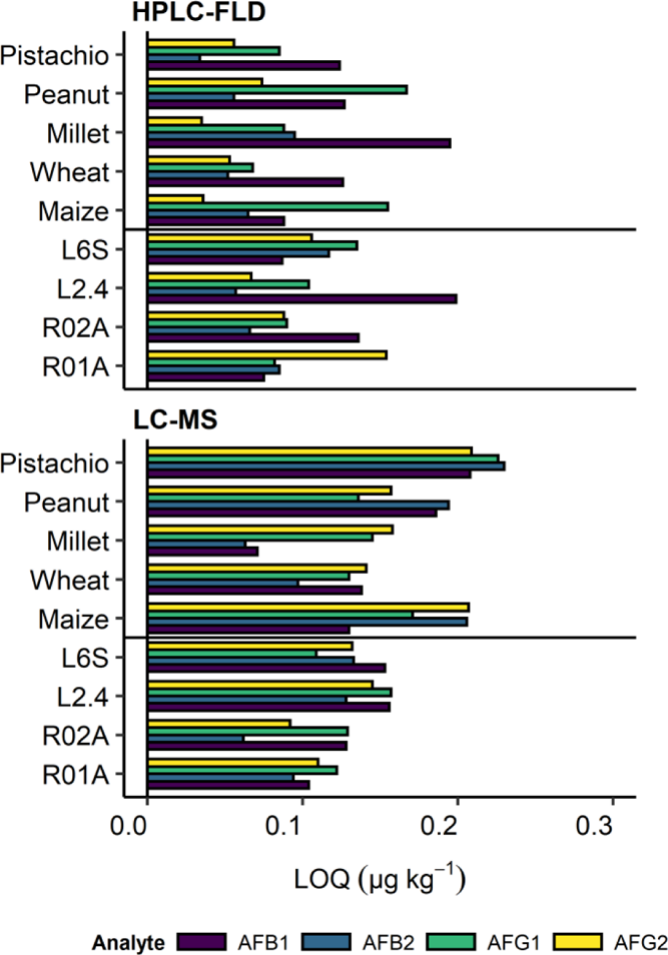
LOQs (method) for investigated AFs measured via LC–MS and HPLC-FLD.

The method’s LOD an LOQ ranged from 0.02 to 0.07 and 0.06 to 0.23 μg kg^–1^ for LC–MS and from 0.01 to 0.07 and 0.04 to 0.23 μg kg^–1^ for HPLC-FLD (Figure 3, Table S1). The method’s LOD and LOQ were significantly (*p* < 0.001, F-ANOVA, d*f* = 1, Table S1 and S3) higher for LC–MS (0.04 ± 0.01 and 0.14 ± 0.04 μg kg^–1^) than for HPLC-FLD (0.03 ± 0.01 and 0.10 ± 0.04 μg kg^–1^). There was no significant (*p* = 0.23 and 0.24, F-ANOVA, d*f* = 1, Tables S1 and S3) difference between values for food and soil matrices. The significant interaction term for LOD (*p* = 0.013, F-ANOVA, d*f* = 1, Tables S1 and S3) and LOQ (*p* = 0.01, F-ANOVA, d*f* = 1, Tables S1 and S3) suggests higher values in food matrices than in soil matrices for the LC–MS, while an opposite pattern was observed for HPLC-FLD.

Interestingly, the method sensitivity in terms of LOD and LOQ was better for the HPLC-FLD compared to the LC–MS. This may be explained by the fact that the lower instrumental sensitivity of the HPLC-FLD (i.e., the analyte concentration-to-signal relationship) was compensated by a much higher injection volume. In LC–MS applications, smaller columns are usually used to enable separations at lower flow rates. Low flow rates are needed to ensure sufficient evaporation of the solvent after leaving the column. With HPLC, larger columns and thus higher injection volumes can be used. In addition, using the on-column focusing technique^43−45^ in which the sample is prepared in a weaker solvent than the mobile phase, it was possible to greatly increase the injection volumes up to 100 μL as compared to the volumes that are usually used for such column dimensions, that is, 8–40 μL as suggested by many manufacturers. Irrespective of the instrumentation, most LOQs were around 10–300 times below the target quantitation levels based on the maximum levels for certain contaminants in foods intended for direct human consumption listed in Commission Regulation (EC) no. 1881/2006.^24^ Only for foods for infants, young children, and for medical use, the LOQs for almost all matrices were above the limit value of 0.1 μg kg^–1^. This threshold may still be achieved by concentrating the extract. However, this would significantly increase the already high matrix effects in foodstuff (LC–MS). Furthermore, it is likely that interfering peaks near the analyte peaks (HPLC-FLD) may broaden considerably due to column overloading and thus lead to an insufficient separation of analyte peaks and interfering peaks. Treatment of the extract by IAC or SPE could simultaneously concentrate the extract and purify it from matrix components so that lower LOQs could be achieved. Altogether, both methods had proven to be suitable for the monitoring of foodstuff for human consumption.

### 3.4 Trueness and Precision

**Figure 4:**
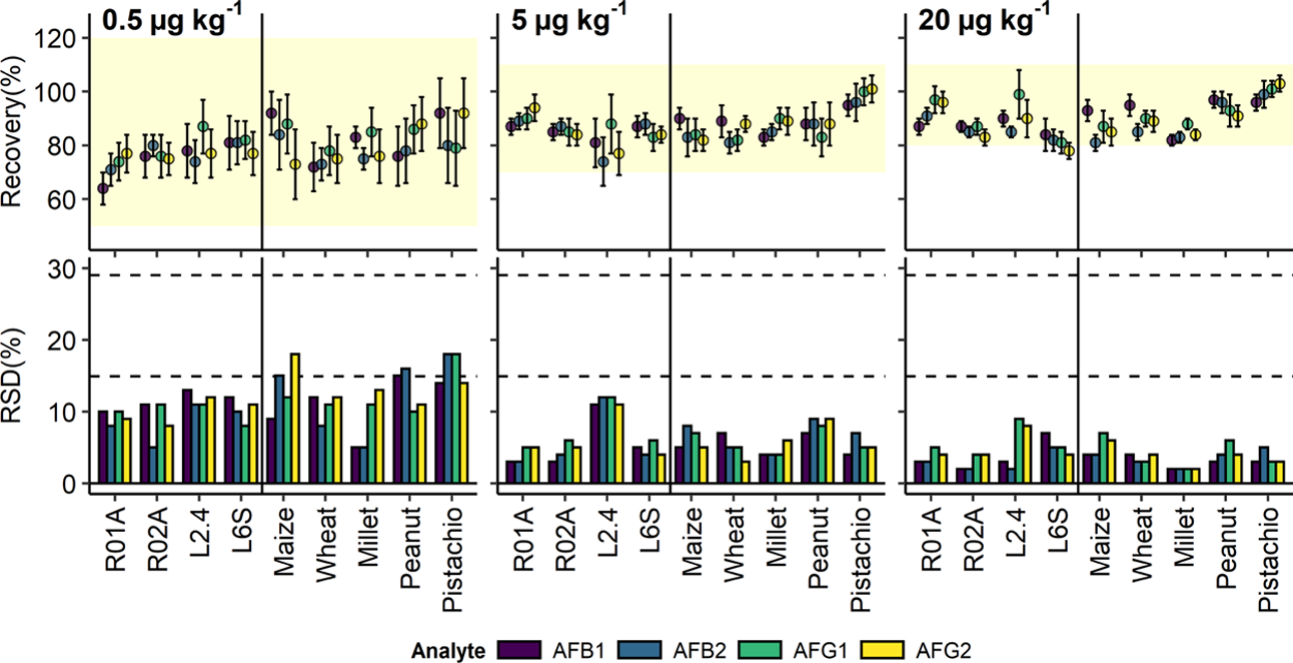
Trueness in terms of mean and standard deviation of spike recovery (top) and relative standard deviation of spike recovery (bottom) for the three fortification levels at 0.5 μg kg^–1^ (left), 5 μg kg^–1^ (center) and 20 μg kg^–1^ (right). Horizontal bands (top) indicate the trueness thresholds set up by the EC of 50–120% for <1 μg kg^–1^ (left), 70–110% for 1–10 μg kg^–1^ (center), and 80–110% for >10 μg kg^–1^ (right). The dashed lines are indication of the maximum recommended and maximum permitted repeatability of 14.52 and 29.04% respectively set by the EC.

The recovery rates ranged from 64 to 92, 74 to 101, and 78 to 103% for the fortification levels of 0.5, 5 and 20 μg kg^–1^, respectively (Figure 4, Table S2). The recovery rates were significantly lower at the lowest fortification level (*p* < 0.001, F-ANOVA, d*f* = 2, Table S2 and S3). In addition, the recovery rates were significantly (*p* = 0.0194, F-ANOVA, d*f* = 2, Table S2 and S3) higher in food matrices than in soil matrices. Furthermore, neither clay content (*p* = 0.507, *t*-test, d*f* = 44, Table S2 and S3) nor *C*_org_ (*p* = 0.494, *t*-test, d*f* = 44, Table S2 and S3) had a significant effect on recovery rates in soil matrices. Overall, the percentage recovery rates were in accordance with the performance criteria imposed by Commission Regulation (EC) no. 401/2006.^24^ Only for the clayey soil (LUFA 6S) at a fortification level of 20 μg kg^–1^, the spike recovery of 78% is slightly lower than the proposed range of 80–110% for levels >10 μg kg^–1^, which may not be problematic since these limits are only valid for food matrices, and so far, no limits are defined for soil matrices. However, the recovery is still fulfilling the limits for soil matrices reported in other guidelines such as the limits of 70–110% defined by the EC in the SANCO/3029/99 rev.411/07/00 guide.^46^ The calculated relative standard deviations of the repeatability were in the range of 2–18% and hence below the maximum permitted relative standard deviation of 29%. Furthermore, 136 out of 144 (≙94%) matrix/fortification level/analyte combinations were below the recommended maximum relative standard deviation of 14.52%. Thereby, all exceeding values originated from the lowest fortification level of 0.5 μg kg^–1^. In general, the RSD was significantly higher at the lowest fortification level (*p* < 0.001, F-ANOVA, d*f* = 2, Tables S2 and S3), but no effect of matrix type (*p* = 0.254, F-ANOVA, d*f* = 1, Table S2 and S3) was observed. Altogether, these results are in line with the regulatory limits, thus confirming a satisfactory performance of method trueness and precision.

### 3.5 Complexity of Soil as a Matrix

Extractions of organic analytes from soil matrices pose an analytical challenge from the point of view of the diverse interaction occurring between soil and pollutants. Nonetheless, the overall recovery rates for all soil matrices and AFs in the present study were by around 11% higher than those presented by Starr and Selim,^8^ despite using a supercritical fluid extraction approach. Strong interactions between AFs and soil organic matter were demonstrated by Schenzel et al.,^10^ with log *K*_OC_ values ranging from 2.80 to 3.46. The authors explained that structural differences between the AFs were responsible for the different *K*_OC_ values. AFs with a double bond such as B1 and G1 resulted in a higher affinity for peat by ∼0.45 log units compared to saturated forms (B2 and G2). Furthermore, van Rensburg et al.^11^ observed strong interactions between AFs and the humic acid oxihumate with binding capacities of 7.4–11.9 mg AFB1 per g of oxihumate over a pH range of 3–7. Clay minerals constitute also effective sites for interactions with AFs.^4,5,12,13^ Results from Goldberg and Angle^9^ suggest that the sorption affinity of AFs to clay minerals may be higher than that to soil organic matter, as a relationship was found between the adsorption coefficient and clay content but not for organic carbon content. Kang et al.^47^ postulated that electron-donor–acceptor interactions between the two electron-rich carbonyl groups (C=O)_2_ in the coumarin structure of the AFs and electron-deficient or positively charged species located at the negatively charged surface of clay minerals (i.e., H^+^ for illite and Ca^2+^ for smectite) are mainly responsible for the strong sorption of AFs to 2:1 clay minerals. However, the analytical method presented in this study was able to overcome these interactions in soils, resulting in suitable values in terms of recovery. The combination of MeCN/H_2_O solvent extraction with ultrasonication was able to successfully extract AFs from soil matrices with clay contents up to 40.9% and organic carbon contents up to 1.99%. Ultrasonication has shown to significantly decrease the particle size of soil agglomerates^48^ and clay minerals^49−51^ and hence increase the surface area, resulting in a more intense contact with the extraction solvent. MeCN is a monopolar solvent that exhibits H-bond acceptor properties (solute H-bond basicity = 0.32)^52^ but insignificant H-bond donor properties (solute H-bond acidity = 0.07)^52^ and hence behaves similar to the carbonyl groups in the coumarin structure of the AFs. Thus, MeCN may competitively displace the AFs from H-bond-accepting sites of the cations, which are located on the negatively charged clay mineral surfaces. Madden and Stahr^6^ used a solvent mixture of similar composition (MeCN/H_2_O, 9:1), but only trace amounts could be recovered. This may be due to a missing ultrasonication step or an insufficient extraction time (4 min). Chloroform, one of the extractants tested by Mertz et al.,^4^ is a monopolar solvent with insignificant H-bond acceptor properties (solute H-bond basicity = 0.02)^52^ and therefore may not be able to compete with AFs for sorption sites. MeOH, the second extractant tested by Mertz et al.,^4^ is a bipolar solvent with both H-bond donor (solute H-bond acidity = 0.43)^52^ and acceptor properties (solute H-bond basicity = 0.47).^52^ Hence, MeOH is also capable of interacting with itself, which may lower the ability to compete with AFs for sorption sites. In addition, the proton acceptor and donor sites are adjacent (within the OH group). Thus, the partial positive charge of hydrogen in the OH-group could hinder the attachment of MeOH to the positively charged cation layer. Angle and Wagner^5^ and Goldberg and Angle^9^ used acetone for extraction experiments, which is a monopolar solvent exhibiting H-bond acceptor properties (solute H-bond basicity = 0.49).^52^ Therefore, acetone can be expected to compete for sorption sites to a similar extent as acetonitrile. While Angle and Wagner^5^ were able to only recover 18% of the spiked amount, Goldberg and Angle^9^ achieved recovery values of around 70%. This discrepancy may be explained by the fact that Goldberg and Angle^9^ presaturated the soil with water before spiking with AFs. Hence, the interaction sites of the clay minerals may already be occupied by water molecules, lowering the affinity of AFs to clay minerals. It has already been shown that hydration of the soil prior to extraction and mixing organic solvents with small amounts of water weakens the interactions of analytes within the soil matrix and makes the pores in the soil more accessible to the extraction solvent.^53^ In the case that only monopolar solvents with H-bond acceptor properties are able to successfully compete for sorption sites with AFs, solvents such as alkenes, alkylaromatic compounds, ethers, ketones esters, and aldehydes could also be suitable candidates for the extraction of AFs from soils.

## 4 Conclusions

For the first time, a simple and reliable method is presented for the quantitative analysis of AFs in soil and food matrices at environmentally relevant concentrations using the same extraction procedure and chromatography, either by HPLC-FLD or LC–MS. Method validation according to the EuraChem guide^23^ indicates the suitability of the method that is also in agreement with precision and recovery requirements of EC Regulation no. 401/2006^24^ for AFB1, AFB2, AFG1, and AFG2 in four soils and five food matrices. Sensitivity allowed quantitative analysis even at trace levels (LOQ between 0.062–0.23 μg kg^–1^ for LC–MS and 0.035–0.231 μg kg^–1^ for the HPLC-FLD. As far as we know, this is the first solvent extraction method presented that achieves suitable and reproducible recovery rates for AFs in soil matrices (in particular in clayey soils) and the first method that does not require extract dilution or cleanup. The necessity for sample purification could be avoided since (i) matrix-matched calibration was capable of compensating matrix effects for LC–MS and (ii) interference peaks could be successfully separated using a weak elution program with a high water content for HPLC-FLD. Furthermore, since the matrix effect was negligible for HPLC-FLD, no matrix-matched calibration would be required and thus, solvent calibration would be sufficient. The absence of a purification step and the possibility to use HPLC-FLD significantly reduces the workload and costs. Therefore, the present method is of particular interest for routine analysis in countries in which levels of AFs may pose a health concern and continuous monitoring is needed in order to assess environmental contamination levels. This simple and rapid method offers also a possibility of capacity building since nonsophisticated analytical tools are needed. However, it remains to be clarified how SPE- or IAC-based purification methods perform in comparison with the presented method. For example, an additional SPE or IAC step could be used to concentrate the extracts, which may not be possible with the present method since a strong peak broadening of the interfering peaks could occur due to column overload. For other food/soil matrices, it may not be possible using the present method, and in this case, sample cleanup techniques such as IAC or SPE are advisable. Finally, the presented method opens up the possibility of reliably assessing the occurrence of AFs in the soil–plant system in agricultural areas. The insights gained from this could help in understanding the factors that lead to preharvest contamination and developing agricultural applications to reduce contamination in the field.

## Supporting Information Available

Validation parameters, mean and relative standard deviation of aflatoxin recoveries, summary of statistic models, HPLC-FLD chromatograms, and solvent and matrix-matched calibration (PDF)

## Abbreviations

AFs: aflatoxins
AFB1: aflatoxin B1
AFB2: aflatoxin B2
AFG1: aflatoxin G1
AFG2: aflatoxin G2
AGC: automatic gain control
CEC: cation exchange capacity
*C*_org_: organic carbon content
HPLC-FLD: high-performance liquid chromatography with fluorescence detection
LC–MS: liquid chromatography–mass spectrometry
LOD: limit of detection
LOQ: limit of quantification
MeCN: acetonitrile
MeOH: methanol
SSE: signal supression/enhancement
USE: ultrasonication-assisted solvent extraction
