# Supplementary material for: Validation of a Simple and Reliable Method for the Determination of Aflatoxins in Soil and Food Matrices
Source: ACS Omega. 2021 Jul 16;6(29):18684–93. doi: 10.1021/acsomega.1c01451 (PMC8319938; doi:10.1021/acsomega.1c01451)
Supplement: Supplementary file 1 — ao1c01451_si_001.pdf [file ao1c01451_si_001.pdf]

Supporting Information

Validation of a simple and reliable method for the determination of aflatoxins in soil and food matrices

Julius Albert[1], Camilla A. More[1], Niklaus R. P. Dahlke[1], Zacharias Steinmetz[2], Gabriele E. Schaumann*[2], Katherine Muñoz[1]

AUTHOR ADDRESS

1 iES Landau, Institute for Environmental Sciences, Group of Organic and Ecological Chemistry, University of Koblenz-Landau, Fortstraße 7, 76829 Landau, Germany

2 iES Landau, Institute for Environmental Sciences, Group of Environmental and Soil Chemistry, University of Koblenz-Landau, Fortstraße 7, 76829 Landau, Germany

Table SI-1. Validation parameters for the mycotoxins investigated in different soil and food matrices using LC-MS and HPLC-FLD. SSE = signal suppression/enhancement, w_i = weighting factor used for weighted calibration, R_{WLS}^2 = coefficient of determination of the weighted calibration, R_{OLS}^2 = coefficient of determination of the unweighted model, $RE_{sum,WLS}$ = sum of relative errors of the weighted calibration model, $RE_{sum,OLS}$ = sum of relative errors of the unweighted calibration model, ΔRE_{sum} = reduction of the sum of relative errors due to application of weighting factor, LOD = limit of detection, LOQ = limit of quantification.

Matrix	Analyte	LC-MS									HPLC-FLD								
		SSE (%)	w_i	R^2_{WLS}	R^2_{OLS}	$RE_{sum,WLS}$ (%)	$RE_{sum,OLS}$ (%)	ΔRE_{sum} (%)	LOD (µg kg^{-1})	LOQ (µg kg^{-1})	SSE (%)	w_i	R_{WLS}^2	R_{OLS}^2	$RE_{sum,WLS}$ (%)	$RE_{sum,OLS}$ (%)	ΔRE_{sum} (%)	LOD (µg kg^{-1})	LOQ (µg kg^{-1})
Refesol 01-A	AFB1	-7	$1/y^2$	0.99	0.997	65	193	-66	0.031	0.104	-1	$1/x^2$	0.967	>0.999	97	145	-33	0.022	0.075
Refesol 01-A	AFB2	-5	$1/y^2$	0.987	0.995	61	99	-38	0.028	0.094	0	$1/x^2$	0.997	0.998	42	390	-89	0.025	0.085
Refesol 01-A	AFG1	-18	$1/y^2$	0.983	0.997	94	517	-82	0.037	0.122	-10	$1/y^1$	0.992	0.994	123	213	-43	0.025	0.082
Refesol 01-A	AFG2	-14	$1/y^2$	0.974	0.995	102	424	-76	0.033	0.11	-12	$1/x^2$	0.985	0.992	103	636	-84	0.046	0.154
Refesol 02-A	AFB1	-7	$1/x^2$	0.989	0.998	85	105	-20	0.039	0.128	-8	$1/y^2$	0.985	0.997	109	591	-82	0.041	0.136
Refesol 02-A	AFB2	-3	$1/x^2$	0.987	0.996	82	194	-58	0.019	0.062	-2	$1/y^2$	0.994	0.999	64	327	-80	0.02	0.066
Refesol 02-A	AFG1	-13	$1/x^2$	0.986	0.996	96	117	-18	0.039	0.129	-14	$1/x^2$	0.991	0.997	78	316	-75	0.027	0.09
Refesol 02-A	AFG2	-12	$1/x^2$	0.986	0.995	89	197	-55	0.028	0.092	-3	$1/y^1$	0.998	0.998	67	306	-78	0.026	0.088
LUFA 2.4	AFB1	-11	$1/x^2$	0.987	0.997	84	360	-77	0.047	0.156	-7	$1/x^2$	0.984	0.979	102	1490	-93	0.06	0.199
LUFA 2.4	AFB2	-12	$1/y^2$	0.993	0.996	64	128	-50	0.038	0.128	-3	$1/y^2$	0.993	0.999	65	397	-84	0.017	0.057
LUFA 2.4	AFG1	-23	$1/y^2$	0.992	0.996	71	265	-73	0.047	0.157	-6	$1/x^2$	0.989	0.987	80	1217	-93	0.031	0.104
LUFA 2.4	AFG2	-20	$1/x^1$	0.998	0.998	67	223	-70	0.044	0.145	0	$1/y^2$	0.997	0.999	44	401	-89	0.02	0.067
LUFA 6S	AFB1	-19	$1/x^2$	0.984	>0.999	82	275	-70	0.046	0.153	-3	$1/x^2$	0.986	0.996	95	651	-85	0.026	0.087
LUFA 6S	AFB2	-23	$1/y^2$	0.984	>0.999	75	202	-63	0.04	0.133	2	$1/y^1$	0.998	>0.999	166	281	-41	0.035	0.117
LUFA 6S	AFG1	-25	$1/y^1$	0.999	>0.999	84	160	-48	0.033	0.109	-14	$1/y^1$	0.996	0.997	178	327	-45	0.04	0.135
LUFA 6S	AFG2	-22	$1/x^2$	0.988	>0.999	65	194	-66	0.04	0.132	-1	$1/y^2$	0.996	0.998	44	537	-92	0.032	0.106
Maize	AFB1	-54	$1/x^2$	0.987	0.992	88	110	-20	0.039	0.13	-7	$1/x^2$	0.972	0.99	121	940	-87	0.026	0.088
Maize	AFB2	-46	$1/x^2$	0.989	0.997	76	208	-64	0.062	0.206	-6	$1/y^2$	0.999	0.999	28	211	-87	0.019	0.065
Maize	AFG1	-49	$1/x^2$	0.991	0.998	78	171	-55	0.051	0.171	2	$1/x^2$	0.988	0.991	80	982	-92	0.047	0.155
Maize	AFG2	-47	$1/x^2$	0.984	0.998	82	214	-62	0.062	0.207	0	$1/x^{0.5}$	0.999	0.999	38	122	-69	0.011	0.036
Wheat	AFB1	-45	$1/x^{0.5}$	0.999	>0.999	130	131	-1	0.041	0.138	-13	$1/x^1$	0.998	0.998	93	343	-73	0.038	0.126
Wheat	AFB2	-41	$1/x^2$	0.994	0.999	52	58	-11	0.029	0.097	-7	$1/x^2$	0.999	>0.999	28	191	-85	0.016	0.052
Wheat	AFG1	-36	$1/x^2$	0.991	0.999	81	277	-71	0.039	0.13	-8	$1/x^2$	0.99	0.989	74	846	-91	0.021	0.068
Wheat	AFG2	-39	$1/x^1$	0.996	0.998	141	365	-61	0.042	0.141	-4	$1/x^2$	0.998	>0.999	30	130	-77	0.016	0.053
Millet	AFB1	-43	$1/x^2$	0.983	0.998	103	232	-56	0.021	0.071	5	$1/y^2$	0.982	0.994	102	757	-87	0.059	0.195
Millet	AFB2	-47	$1/x^2$	0.987	0.995	83	87	-4	0.019	0.063	5	$1/x^2$	0.999	0.999	25	235	-89	0.028	0.095
Millet	AFG1	-46	$1/x^2$	0.986	0.994	100	360	-72	0.043	0.145	0	$1/x^2$	0.996	0.994	49	672	-93	0.027	0.088

Millet	AFG2	-43	$1/x^2$	0.989	0.994	84	314	-73	0.047	0.158	3	$1/y^2$	0.989	0.999	75	137	-45	0.01	0.035
Peanut	AFB1	-40	$1/y^2$	0.98	0.991	104	159	-35	0.056	0.186	-6	$1/x^2$	0.975	0.989	131	1383	-91	0.038	0.127
Peanut	AFB2	-47	$1/x^2$	0.988	0.998	85	104	-18	0.058	0.194	-4	$1/x^2$	0.992	>0.999	54	148	-64	0.017	0.056
Peanut	AFG1	-50	$1/x^2$	0.986	0.997	90	165	-45	0.041	0.136	-6	$1/x^2$	0.993	0.993	68	787	-91	0.05	0.167
Peanut	AFG2	-43	$1/x^2$	0.986	0.995	90	187	-52	0.047	0.157	-4	$1/y^1$	1	>0.999	34	92	-63	0.022	0.074
Pistachio	AFB1	-43	$1/y^2$	0.985	0.998	35	240	-86	0.062	0.208	9	$1/x^2$	0.966	0.997	130	732	-82	0.037	0.124
Pistachio	AFB2	-48	$1/y^2$	0.994	0.999	32	214	-85	0.069	0.23	-1	$1/x^2$	0.996	0.998	53	388	-86	0.01	0.034
Pistachio	AFG1	-52	$1/y^2$	0.997	0.999	20	173	-88	0.068	0.226	4	$1/x^2$	0.953	0.999	144	632	-77	0.026	0.085
Pistachio	AFG2	-53	$1/y^2$	0.991	0.999	32	237	-86	0.063	0.209	5	$1/y^2$	0.996	0.998	58	534	-89	0.017	0.056

Table SI-2. Mean and relative standard deviation (in brackets) of recoveries for the mycotoxins investigated in different soil and food matrices at three fortification levels (N=10 each) and overall (N=30).

Matrix	Analyte	Recovery			
		0.5 μg kg^{-1}	5 μg kg^{-1}	20 μg kg^{-1}	Overall
Refesol 01-A	AFB1	64(10)	87(3)	87(3)	80(15)
Refesol 01-A	AFB2	71(8)	89(3)	91(3)	84(12)
Refesol 01-A	AFG1	74(10)	90(5)	97(5)	87(13)
Refesol 01-A	AFG2	77(9)	94(5)	96(4)	89(12)
Refesol 02-A	AFB1	76(11)	85(3)	87(2)	83(8)
Refesol 02-A	AFB2	80(5)	87(4)	85(2)	84(5)
Refesol 02-A	AFG1	76(11)	85(6)	87(4)	83(9)
Refesol 0-2A	AFG2	75(8)	84(5)	83(4)	81(8)
LUFA 2.4	AFB1	78(13)	81(11)	90(3)	83(11)
LUFA 2.4	AFB2	74(11)	74(12)	85(2)	78(11)
LUFA 2.4	AFG1	87(11)	88(12)	99(9)	92(12)
LUFA 2.4	AFG2	77(12)	77(11)	90(8)	81(12)
LUFA 6S	AFB1	81(12)	87(5)	84(7)	84(8)
LUFA 6S	AFB2	81(10)	88(4)	82(5)	84(8)
LUFA 6S	AFG1	82(8)	83(6)	81(5)	82(7)
LUFA 6S	AFG2	77(11)	84(4)	78(4)	80(7)
Maize	AFB1	92(9)	90(5)	93(4)	92(6)
Maize	AFB2	84(15)	83(8)	81(4)	83(10)
Maize	AFG1	88(12)	84(7)	87(7)	86(9)
Maize	AFG2	73(18)	82(5)	85(6)	80(12)
Wheat	AFB1	72(12)	89(7)	95(4)	85(14)
Wheat	AFB2	73(8)	81(5)	85(3)	79(8)
Wheat	AFG1	78(11)	82(5)	90(3)	83(9)
Wheat	AFG2	75(12)	88(3)	89(4)	84(10)
Millet	AFB1	83(5)	83(4)	82(2)	83(4)
Millet	AFB2	75(5)	85(4)	83(2)	81(7)
Millet	AFG1	85(11)	90(4)	88(2)	88(7)
Millet	AFG2	76(13)	89(6)	84(2)	83(10)
Peanut	AFB1	76(15)	88(7)	97(3)	87(13)
Peanut	AFB2	78(16)	88(9)	96(4)	88(13)
Peanut	AFG1	86(10)	83(8)	93(6)	87(9)
Peanut	AFG2	88(11)	88(9)	91(4)	89(8)
Pistachio	AFB1	92(14)	95(4)	96(3)	94(8)
Pistachio	AFB2	80(18)	96(7)	99(5)	92(14)
Pistachio	AFG1	79(18)	100(5)	101(3)	93(15)
Pistachio	AFG2	92(14)	101(5)	103(3)	99(10)

Table SI-3. Summary of statistic models: Effects of weighting (Weighting), intrument (LC-MS, HPLC-FLD), matrix type (soil, food), fortification level (low, medium, high) and their interactions on coefficient of determination (R_{adj}^2), sum of percentage relative error (RE_{sum}(%)), matrix effect (|SSE|), limit of detection and quantification (LOD, LOQ), relative spike recovery (Recovery) and relative standard deviation of spike recovery (RSD_r).

Parameter	Predictor	DF	Test statistics	Value	p
R^2_{adj}	Weighting	79	Paired T-Test	7.52	<0.001
RE_{sum}(%)	Weighting	79	Paired T-Test	-8.33	<0.001
|SSE|	Instrument	1	F-ANOVA	512.1	<0.001
|SSE|	Matrix type	1	F-ANOVA	165.6	<0.001
|SSE|	Instrument: Matrix type	1	F-ANOVA	174.9	<0.001
LOD	Instrument	1	F-ANOVA	24.07	<0.001
LOD	Matrix type	1	F-ANOVA	1.43	0.24
LOD	Instrument: Matrix type	1	F-ANOVA	6.58	0.01
LOQ	Instrument	1	F-ANOVA	24.19	<0.001
LOQ	Matrix type	1	F-ANOVA	1.49	0.23
LOQ	Instrument: Matrix type	1	F-ANOVA	7.01	0.01
Recovery	Matrix type	1	F-ANOVA	5.62	0.02
Recovery	Fortification level	2	F-ANOVA	21.36	<0.001
Recovery	Matrix type: Fortification level	2	F-ANOVA	0.44	0.44
Recovery	Clay content	44	T-Test	-0.67	0.51
Recovery	CEC	44	T-Test	-0.89	0.38
Recovery	C_{org}	44	T-Test	-0.69	0.49
RSD_r	Matrix type	1	F-ANOVA	1.31	0.25
RSD_r	Fortification level	2	F-ANOVA	91.49	<0.001
RSD_r	Matrix type: Fortification level	2	F-ANOVA	4.99	0.01

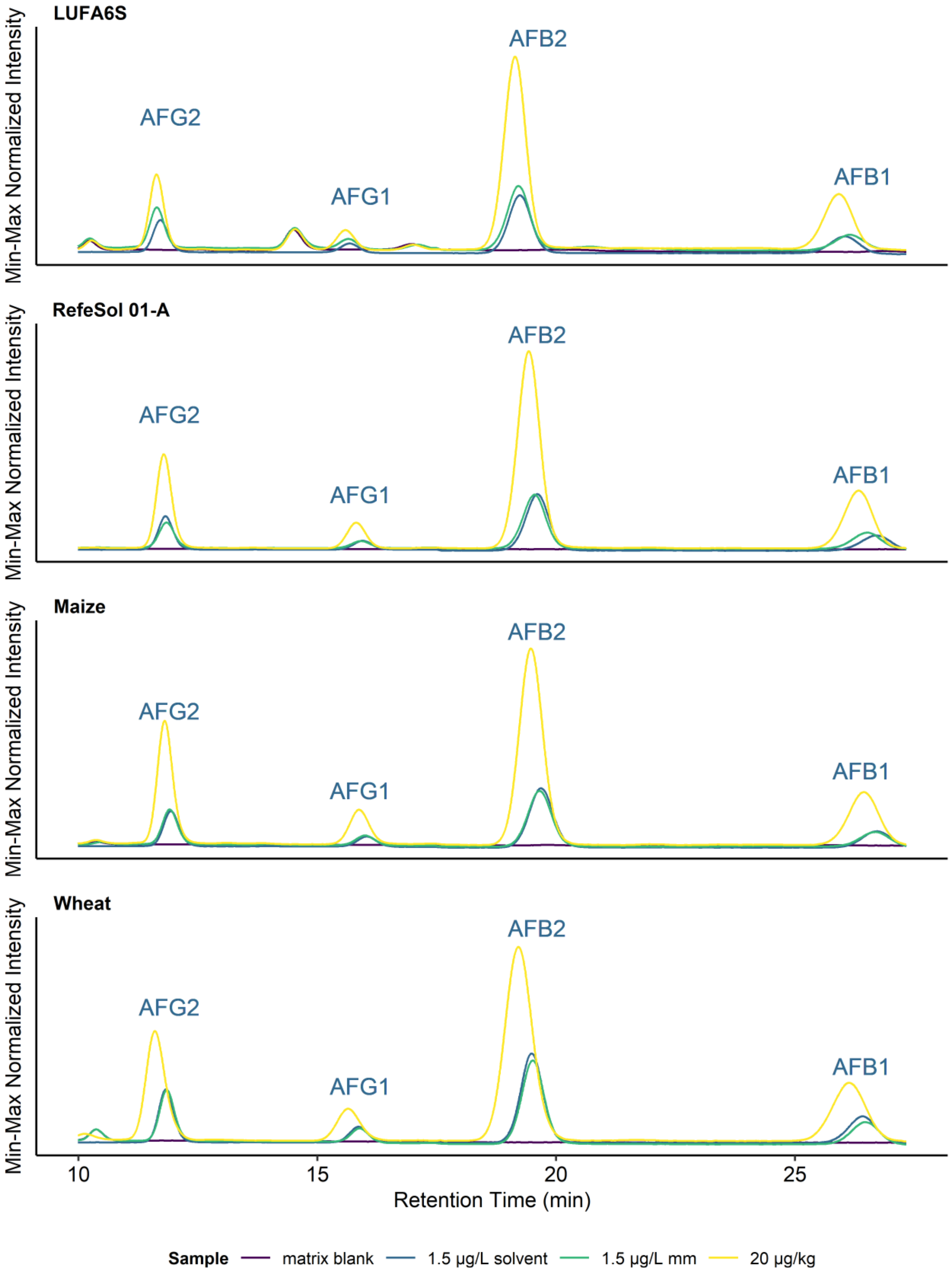

Figure SI-1. HPLC-FLD chromatograms obtained from injection of spiked samples (fortification level 20 µg kg^{-1}), sample blank and matrix standard solution (1.5 µg L^{-1}) of the soils Refesol 01-A and LUFA6 and food matrices maize and wheat (highlighted by different colors).

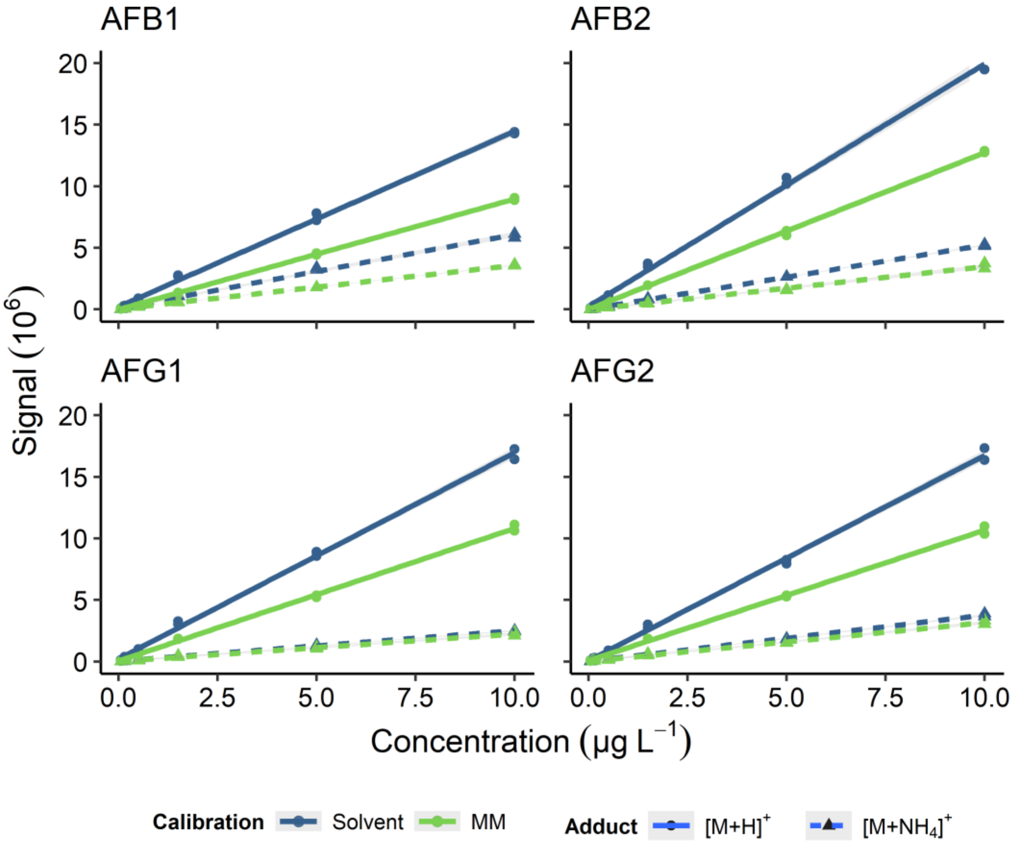

Figure SI-2. Solvent (green) and matrix-matched (blue) calibration for Wheat samples performed with the $[M+H]^+$ (solid) and $[M+NH4]^+$ (dashed) adducts
